# Thermal conductivity reduction in silicon fishbone nanowires

**DOI:** 10.1038/s41598-018-22509-0

**Published:** 2018-03-13

**Authors:** Jeremie Maire, Roman Anufriev, Takuma Hori, Junichiro Shiomi, Sebastian Volz, Masahiro Nomura

**Affiliations:** 1 Institute of Industrial Science, The University of Tokyo, Tokyo, 153-8505 Japan; 2 Laboratory for Integrated Micro Mechatronic Systems/National Center for Scientific Research-Institute of Industrial Science (LIMMS/CNRS-IIS), The University of Tokyo, Tokyo, 153-8505 Japan; 3 Department of Mechanical Engineering, The University of Tokyo, 7-3-1 Hongo, Bunkyo, Tokyo, 113-8656 Japan; 4 Center for Materials Research by Information Integration, National Institute for Materials Science, 1-2-1 Sengen, Tsukuba, Ibaraki, 305-0047 Japan; 5 PRESTO, Japan Science and Technology Agency, Saitama, 332-0012 Japan

## Abstract

Semiconductor nanowires are potential building blocks for future thermoelectrics because of their low thermal conductivity. Recent theoretical works suggest that thermal conductivity of nanowires can be further reduced by additional constrictions, pillars or wings. Here, we experimentally study heat conduction in silicon nanowires with periodic wings, called fishbone nanowires. We find that like in pristine nanowires, the nanowire cross-section controls thermal conductivity of fishbone nanowires. However, the periodic wings further reduce the thermal conductivity. Whereas an increase in the wing width only slightly affects the thermal conductivity, an increase in the wing depth clearly reduces thermal conductivity, and this reduction is stronger in the structures with narrower nanowires. Our experimental data is supported by the Callaway-Holland model, finite element modelling and phonon transport simulations.

## Introduction

Thermal transport in low dimensional and nanostructured materials has attracted high attention over the past decades, in particular with regards to promising prospects in thermoelectric energy generation^1^, including the possibility of using the wave properties of phonons, which can be relevant at cryogenic temperatures^2,3^. Nonetheless, the main impact of semiconductor nanostructures on thermal transport comes from scattering of the heat carriers — phonons. In that regard, semiconductor nanowires (NWs) are the focus of much attention^4–6^ and remain to date one of the most promising building blocks for thermoelectric^6–9^ and other microelectronic devices. Generally, the thermal conductivity of NWs depends on the diameter^4,10–14^ and surface properties^4,7,14–18^, because heat conduction in nanostructures is suppressed by diffuse scattering of phonons on the surfaces^19,20^. For example, a few experimental works^21,22^ have demonstrated a reduction of thermal conductivity in corrugated silicon NWs due to the limited phonon mean free path^21,22^. To further enhance this surface scattering, theoretical works^23–26^ proposed various diameter-modulated NWs and found that heat conduction is strongly suppressed in these structures. Not only is it possible to reduce thermal conductivity proportionally to the ratio between the corrugation and the central constriction, but this reduction can be larger than an order of magnitude at room temperature for structures of a couple of nanometers in width^25^. Despite the difference in scales, lattice dynamics^25^, Monte-Carlo simulations^23,27^, and mixed calculations^24^ agree that reducing the width of the central constriction or increasing the depth of the corrugation reduces thermal conductivity. Thus, modification of the sidewall shape of NWs is a promising approach to further thermal conductivity reduction.

In this work, we systematically study heat conduction in NWs with periodic wings, called hereafter fishbone NWs, which have features of both NWs and phononic crystals. First, we find that thermal conductivity is reduced as the central part — the neck — becomes smaller. Next, we demonstrate that wing size in the direction parallel to heat flux does not strongly affect heat conduction, whereas wing size in the direction perpendicular to the heat flux can significantly reduce thermal conductivity. We explain this reduction by the trapping and backscattering of phonons in the wings. Overall, we experimentally demonstrate that the transient behaviour of the fishbone NWs follow the mass contrast, and that thermal conductivity and thermal relaxation rates can be reduced at room temperature by more than 20% and 35%, respectively.

## Fabrication and Measurements

**Figure 1:**
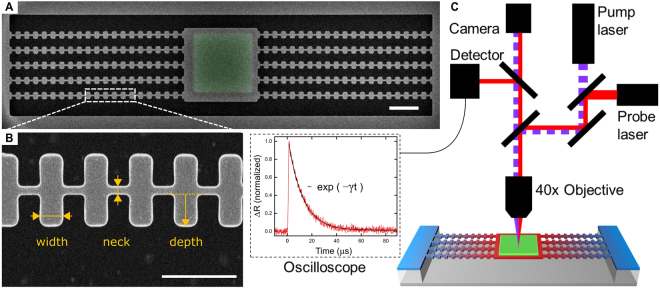
Samples and experimental setup. (**A**) SEM image of a suspended fishbone nanostructure. (**B**) Close-up top view of fishbone NWs with relevant dimensions; Scale bars are 2 µm in (**A**) and 500 nm in (**B**). (**C**) Schematic of µ**-**TDTR setup, with recorded signal and exponential fit.

All samples are fabricated on a (100) silicon-on-insulator (SOI) wafer. The nanostructures are processed on the 145 nm-thick undoped upper single-crystalline silicon layer. 4 × 4 µm^2^ squares are drawn by electron beam lithography and then 125-nm-thick aluminum pads are deposited via electron beam assisted metal evaporation. The shape of the fishbone NWs are drawn by electron beam lithography around the existing aluminum pads followed by a transfer to the silicon layer by means of an inductively coupled plasma reactive-ion etching (Oxford instruments PlasmaLab 100) using a mixture of SF_6_ and O_2_. To suspend the samples, the buried oxide layer is subsequently removed with hydrofluoric acid in vapor phase. Figure 1A and B show scanning electron microscope (SEM) images of a complete structure and a close-up view of a fishbone NW with relevant dimensions.

The thermal properties of all samples are measured using the micro time domain thermoreflectance (µ-TDTR) technique^10,20^, which we have developed for the measurement of suspended nanostructures. A schematic of the setup is shown in Fig. 1C. The aluminum pad in the center of the structures serves as a heater and sensor as its temperature is monitored by a continuous-wave laser (785 nm). After the pad is heated by the pulsed laser (642 nm), the heat flows through the fishbone NWs. The corresponding cooling of the central pad is recorded as a decay of the change in reflectance, which can be fitted by an exponential function in spite of the ballisticity of phonons and intricate geometry of the structure. The decay curve is approximated by a single-parameter exponential decay curve *exp*(−*t*/τ), where τ is the characteristic decay time inversely proportional to the thermal conductance. To extract the thermal conductivity, we use a three dimensional finite element model, which virtually reproduces our experiment^28,29^. The uncertainty on the measurement of the structure dimensions (±3 nm) results in an error in thermal conductivity of less than ±5%. More details of the fabrication method and measurement system are provided in the methods section and in our previous works^3,20,28–30^.

## Results and Discussion

**Figure 2:**
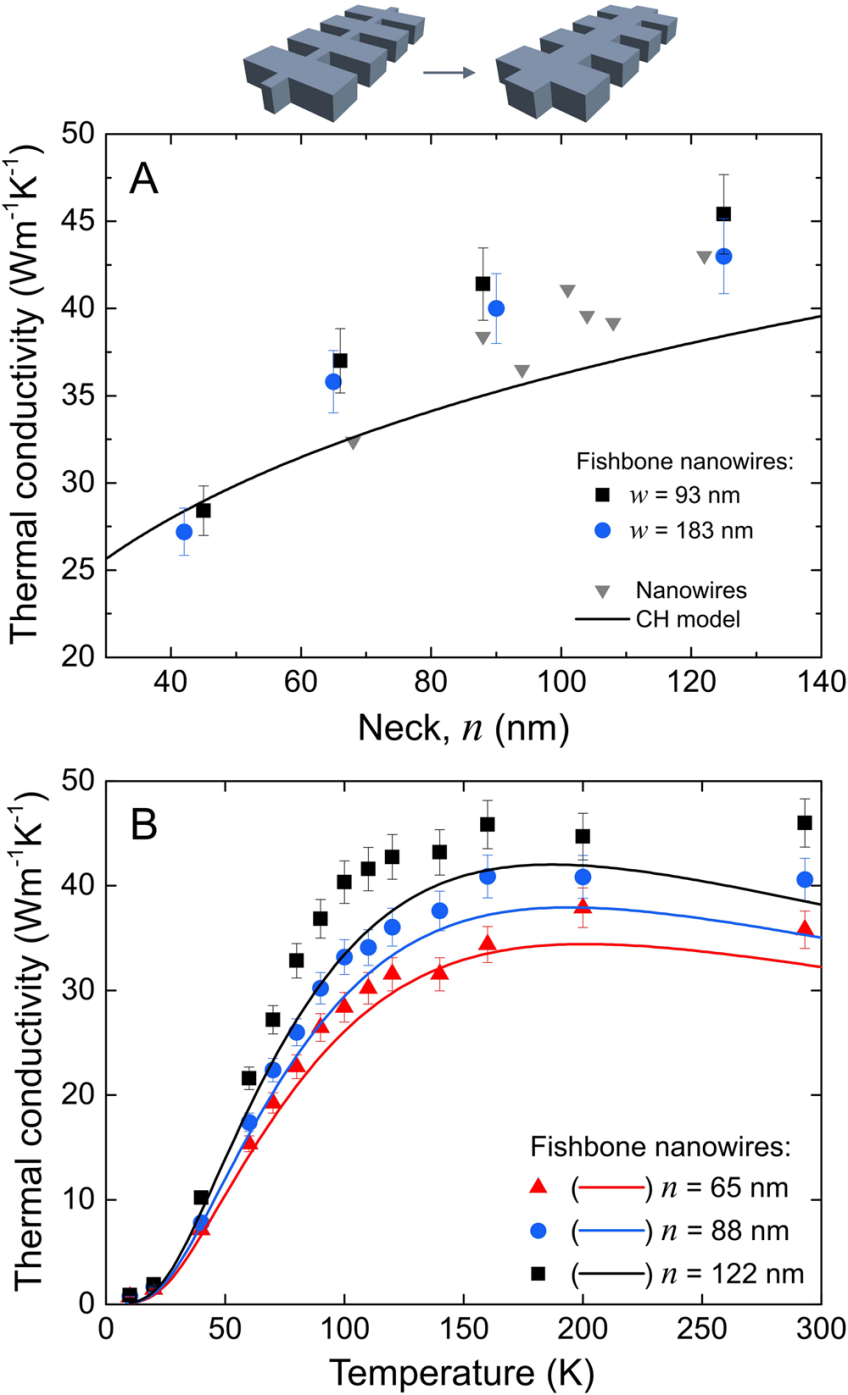
Neck dependence of thermal conductivity. (**A**) Dependence of thermal conductivity at room temperature on the neck width (*n*) for NWs and two sets of fishbone NWs (*d* = 190 nm, *w* = 93 and 183 nm). (**B**) Thermal conductivity in the 10–300 K temperature range for fishbone NWs with the necks of 65, 88 and 122 nm (*d* = 190 nm and *w* = 93 nm). Predictions of Callaway-Holland model are shown by solid lines.

To understand the mechanisms impacting thermal transport in these structures, we investigate the impact of the geometry on thermal conduction by comparing samples with different necks (*n*), wing widths (*w*), and wing depths (*d*) (Fig. 1B). From a geometrical point of view, fishbone NWs are NWs with wings every 300 nm. Thus, we can expect that the neck controls heat conduction^23,27,31,32^, just as it does in NWs^10,11^. Figure 2A shows that the thermal conductivity of the fishbone NWs indeed decreases as the neck is decreased and the absolute values are similar to those of NWs without wings. This dependence is in agreement with the Callaway-Holland model for NWs^10^, as shown in Fig. 2A. In this model, the boundary scattering part of the phonon relaxation time is proportional to the limiting dimension of the structure, which in this case is proportional to the NW neck^10^. For narrower fishbone NWs, the decrease in thermal conductivity seems more pronounced than that of the Callaway-Holland model. As no data is available for NWs, the discrepancy can either stem from a difference between the theoretical model and experimental measurements, or from a specific attribute of fishbone NWs. Since the theoretical model uses one limiting dimension as the parameter to calculate the boundary scattering term, it cannot properly render the geometric complexity of fishbone NWs. Furthermore, it seems that wide NWs (120 nm) have higher thermal conductivity than the model, whereas narrower ones (70 nm) have a similar thermal conductivity as the model, pointing again to the simplicity of the model being unable to fit the physical phenomenon perfectly. Last, as will be shown below, the impact of the wings, i.e., a decrease in thermal conductivity, becomes stronger as the neck becomes narrower, thus partially explaining the trend observed.

As temperature is decreased, the thermal conductivity of fishbone NWs decreases, as shown in Fig. 2B. This behaviour is similar to that of NWs^10^ and is also in good agreement with the Callaway-Holland model^10,33^. The lower thermal conductivity at low temperatures stems from the reduced specific heat and enhanced surface scattering. Indeed, as temperature decreases, the frequencies of phonons contributing to heat transport decrease, hence their mean free path (MFP) in the bulk increases^34–36^, but remains strongly limited in the nanowires^10,11,21,37^. Therefore, the suppression of thermal conductivity by surface scattering is stronger at low temperatures. These results show that the neck is a key parameter in tuning thermal conductivity, as also predicted by Monte Carlo simulations of NWs with constrictions^23,27^.

**Figure 3:**
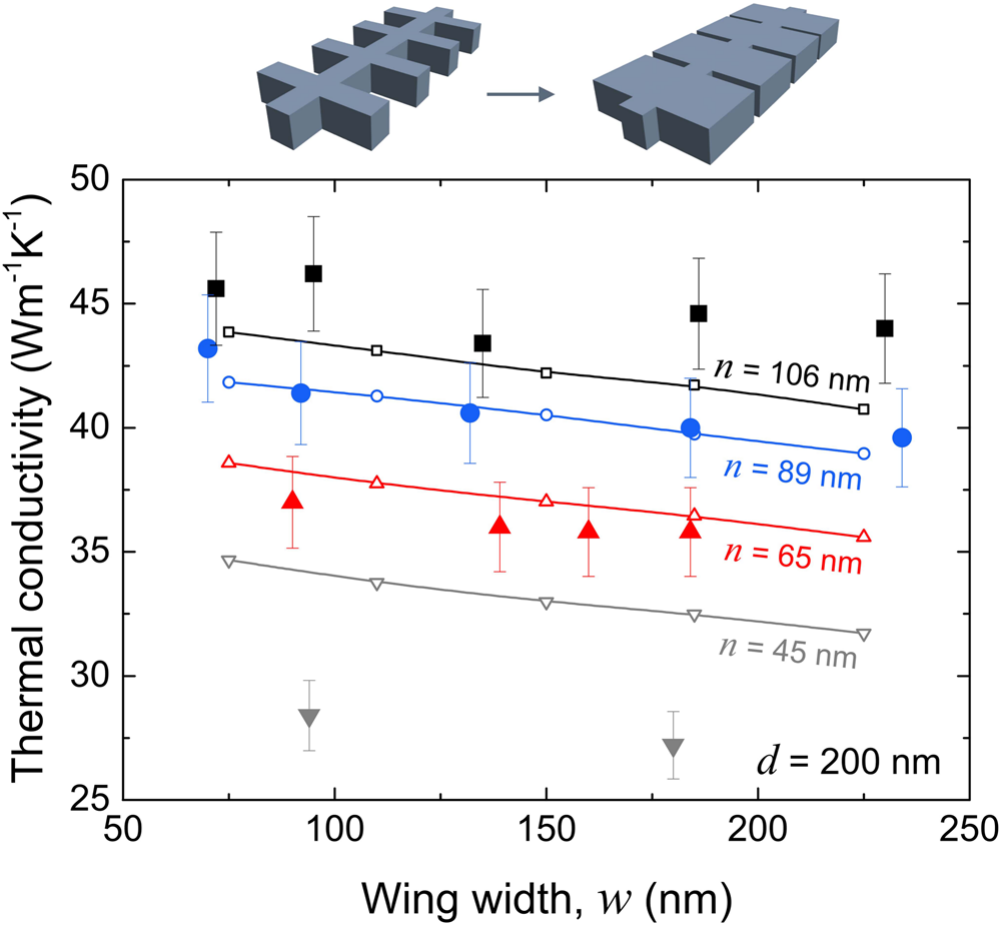
Wing width dependent thermal conductivity. Thermal conductivity at 300 K as a function of wing width (*w*) for samples with different necks (*n*) and the same wing depth (*d* = 200 nm). Results of phonon transport simulations are shown by solid lines with dots.

Next, we fix the neck of the structure and study the impact of the wing width on the thermal conductivity, keeping constant the wing depth (*d* = 200 nm) for all samples. Interestingly, although an increase in the wing width increases thermal conductance (*G* ~ τ^−1^), due to the increase of material volume, the thermal conductivity actually slightly decreases, as shown in Fig. 3. This dependence on the wing width cannot be understood within the Callaway-Holland model because of the complicated geometry. Thus, we perform phonon transport simulations^28,38,39^ of the phonon transport in this geometry, with diffuse surface scattering conditions. The simulation details are provided in the methods section. Figure 3 shows that the simulation results agree with our experimental data and confirm that the wing width does not significantly change thermal conductivity. The agreement with the phonon transport simulations also indicates that the width dependence of thermal conductivity can be explained by phonon boundary scattering alone. Indeed, the increase in the wing width increases the probability for a phonon to enter the wing and thus be scattered backwards and trapped. Interestingly, the experimental data for samples with the smallest neck (*n* = 45 nm) is significantly below the predictions of the phonon transport simulations. This may indicate the narrow regions generate the confinement effects, which are not taken into account in the simulation, as only boundary scattering effects are considered (see Methods).

**Figure 4:**
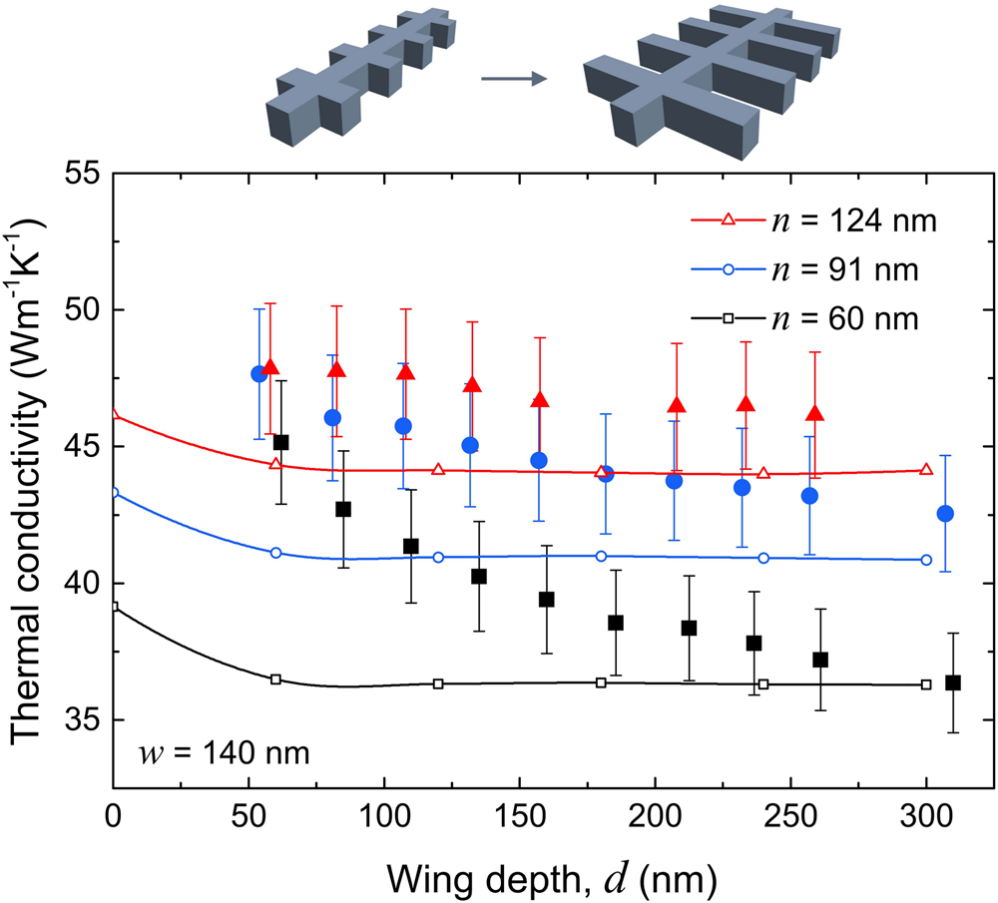
Wing depth dependence of thermal conductivity. Thermal conductivity at room temperature as a function of wing depth (*d*) for three sets of samples with necks of 60 (squares), 91 (circles) and 124 nm (triangles), and the same wing width (*w* = 140 nm). Results of phonon transport simulations are shown by the solid lines with dots.

Next, we fix the wing width (*w* = 140 nm) and study the impact of wing depth (*d*). We measure three different sets of samples with the necks of 60, 91 and 124 nm and wing depth in the 50–300 nm range for each neck. The measurement results (Fig. 4) show that an increase in wing depth causes a reduction in thermal conductivity and this reduction strengthens as the neck narrows. This might be explained by the fact that phonons have lower probability to enter the next unit due to narrower neck and thus stay longer in the wings.

Similar dependence on the wing depth has been predicted by some theoretical works. Lattice dynamics simulations^25^ showed a decrease in thermal conductivity with wing depth in atomic scale structures and explained this reduction by the redistribution of the phonon energy spectrum and reduced phonon group velocities. Monte-Carlo simulations^27^ also showed that thermal conductivity decreased with the wing depth and that the relative decrease is stronger for smaller necks.

Figure 4 also shows our phonon transport simulation data (lines) alongside the experimental results. The values of thermal conductivity for NWs agree within ±2 Wm^−1^K^−1^ with similar Monte-Carlo simulations by Verdier *et al*.^26^. As the wings form and deepen, our simulations predict a slight reduction of thermal conductivity, but this tendency saturates for wings deeper than 100 nm. Moreover, the dependence is much weaker than that observed experimentally. These results suggest that some other mechanism, not taken into account in the phonon transport simulations, affects phonon transport. For example, Nika *et al*.^25^ proposed phonon trapping in the wings as a mechanism that hinders heat transport and is linked to the depth of the wings. They also showed that specular reflections induce a stronger reduction of heat flux in the fishbone NWs. This phenomenon suggests that phonons become trapped in the wing and cannot resume their path through the structure.

**Figure 5:**
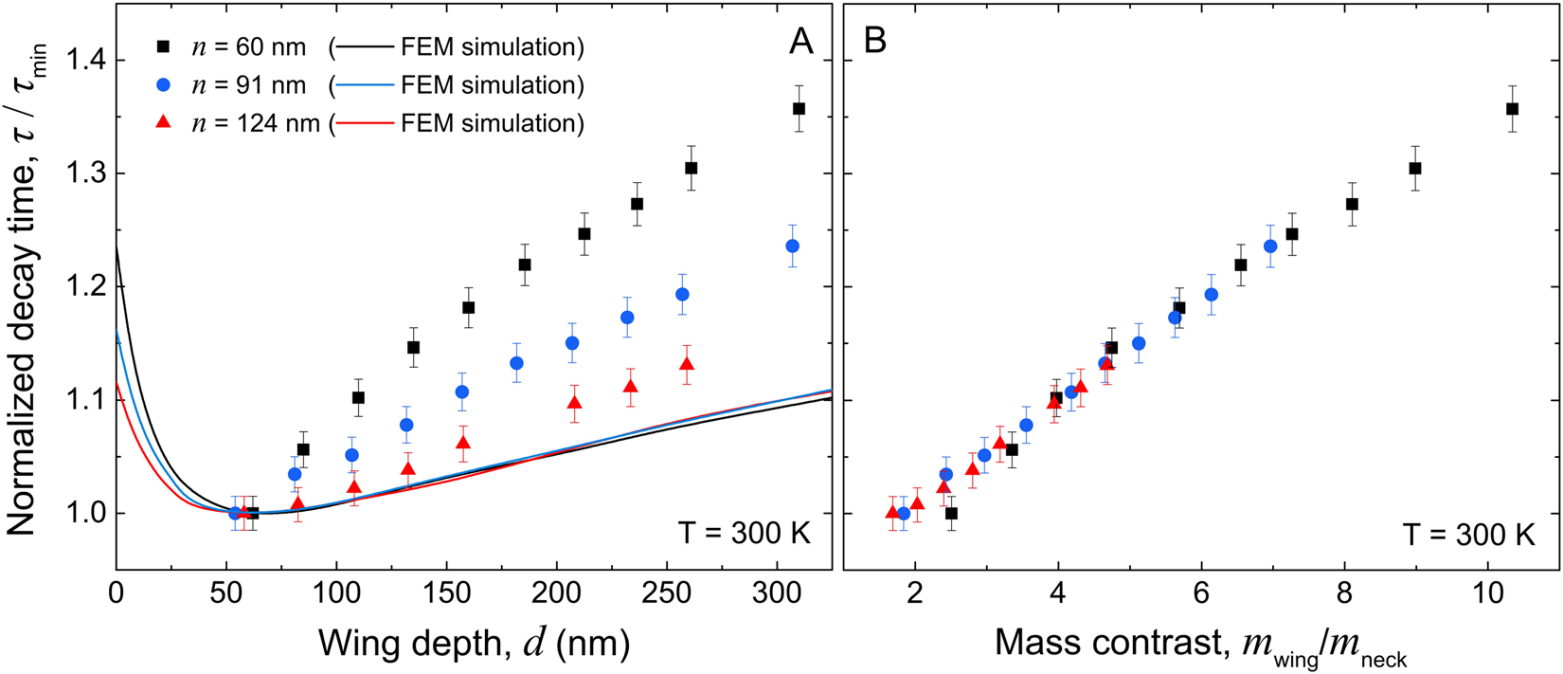
Wing depth dependence of thermal dissipation. Measured room temperature decay times normalized to that of the structure with *d* ≈ 50 nm as a function of wing depth (**A**) and mass contrast (**B**) for three sets of samples with necks of 60 (squares), 91 (circles) and 124 nm (triangles) and the same wing width (*w* = 140 nm). Results of FEM simulations are by lines in panel (A).

To better understand the observed wing depth dependence, we analyze the experimental data obtained in the transient regime. Indeed, whereas thermal conductivity is calculated as a steady state property — the temperature gradient is fixed — the experiment is characterized by the heat dissipation time (or decay time, τ), which reflects the transient behaviour of the system. We first use a simulation model based on finite element method (FEM) to analyze our experiment in the Fourier law approximation. Figure 5A shows that introducing short (<50 nm) wings to a NW causes faster heat dissipation, until a critical depth of around 50 nm is reached, after which a further increase in wing depth increases the decay time, independently of the neck. The increase in the decay time can be understood considering a solution of a classical heat transport equation: *exp*(− *t*/*R·C*), where *R* is the thermal resistance and *C* is the heat capacity. From this form of solution, we can see that our experimental decay time (τ) is simply proportional to *R·C*. In turn, heat capacity is proportional to the volume of the structure, which increases with the wind depth. Hence the decay time is proportional to the wing depth. Our experiments, however, show that the increase is stronger than predicted by the FEM simulations, and that this increase strengthens as the NW narrows. We find that the normalized experimental decay time (τ/τ_min_) is proportional to the mass contrast (*m*_wing_/*m*_neck_), and that the proportionality is the same regardless of the neck, as shown in Fig. 5B.

The most probable explanation for the discrepancies between our experimental results and simulations stems from elements not taken into account in our simulations, such as the group velocity of phonons and the energy distribution. Indeed, the FEM analysis uses simple Fourier law whereas the phonon properties in our phonon transport simulations are calculated from the bulk. Thus, modifications of the group velocities and redistribution of the energy spectrum, as suggested by Nika *et al*.^25^, are not taken into account. This phenomenon, that they call phonon trapping, is more efficient for narrow necks and deeper wings, as is observed in our experiments. Molecular dynamics simulations were used in two different works by Xiong *et al*.^40^ and Ma *et al*.^41^ to arrive to similar conclusions. Indeed, Xiong *et al*. observed a reduction in the group velocity of phonons, but also of the mean free path, stemming from a hybridization of propagating modes with resonant ones. Ma *et al*., working with nanowires cage structures, showed that localization occur at the junction of perpendicular nanowires, with local resonances and hybridization also inducing a strong reduction in thermal conductivity, and demonstrated that this was a local effect, independent of the periodicity. However, as our experiment do not directly prove these theories, further investigations are needed to experimentally clarify the role of the phonon dispersion relation on thermal transport in these structures.

In conclusion, we investigate heat conduction in silicon fishbone NWs at room temperature and report series of thermal conductivity data measured for various structural parameters such as neck size, wing depth, and wing width. Phonon transport simulations capture the steady state behavior of our structures, whereas transient FEM simulations show an impact of geometry on the thermal relaxation time. We show experimentally that the thermal conductivity can be tuned by adjusting the shape of the structures: in the range covered by our experiment, an increase in mass contrast decreases the thermal conductivity by 20%. Since this decrease can be more than three times stronger than predicted by simulations, our experimental results suggest the presence of some other mechanism of thermal conductivity reduction, which is not captured by these simulations. We believe that the difference stems from a transient mechanism and can be partially attributed to the trapping of phonons in the wings and further experiments should clarify the additional mechanisms. However, it is clear that our fishbone NWs have an additional degree of freedom in heat conduction control as compared with pristine NWs. Additionally, since the total volume increases but the neck remains constant as the wings become bigger, the electrical conductivity will not be reduced. Thus, fishbone NWs are promising for thermoelectric applications.

## Methods

### Thermal conductivity measurements

The optical measurement system used in this work is an originally developed micro time-domain thermoreflectance (µ-TDTR) system, using two separate laser diodes. The pulsed pump and continuous probe laser beams have wavelengths of 642 nm and 785 nm, respectively. The laser beams are focused through a 40× microscope lens with a numerical aperture of 0.6 onto the aluminum pad which serves as a heater and sensor. The sample is mounted in a He-flow cryostat (Oxford Instruments) and the pressure is kept low enough to neglect all convection. Radiation can also be neglected. The reflected probe beam is then detected by a silicon photodiode with a bandwidth of 200 MHz. The average of 10^4^ waveforms is then calculated by an oscilloscope with bandwidth of 1 GHz (Tektronix), before being further box-averaged to improve the signal-to-noise ratio. The heat provided by the lasers to the aluminum pads can dissipate through the structures under study only. The measurement output is proportional to a temperature and reveals a decaying trend of the form *exp*(−*t/*τ) with τ the decay time.

The experiment is then reproduced in a 3D Finite Element Model. Each structure parameters are measured from SEM images and used to recreate this structure in 3D with Comsol Multiphysics. The aluminium pad is heated by an inward heat flux of identical duration to the laser pulse in the experiment. The temperature of the aluminium pad’s centre is then monitored until it is back to the bath temperature. This simulation is performed for different values of the thermal conductivity, whereas all other parameters are fixed, and the decay time is extracted for each simulation. We observe that a single exponential decay fits well on all nanowires and fishbone decay curves. Thermal decay times are then plotted as a function of the thermal conductivity and the experimental measurement is fitted on this curve, thus giving the experimental thermal conductivity. Further details about the experimental setup and thermal conductivity extraction can be found in our previous work^30^.

### Phonon transport simulations

The heat conduction in fishbone NWs is simulated using ray tracing method and assuming the bulk properties of phonons, obtained from the first principle calculations. In the nanostructures, the bulk phonon MFP are shortened by boundary scattering at the sidewalls of the nanostructures, and the effective MFP (Λ_eff_) can be expressed via Matthiessen’s rule as ${{\rm{\Lambda }}}_{{\rm{e}}{\rm{f}}{\rm{f}}}^{-1}={{\rm{\Lambda }}}_{{\rm{b}}{\rm{u}}{\rm{l}}{\rm{k}}}^{-1}\,+{{\rm{\Lambda }}}_{{\rm{b}}{\rm{d}}{\rm{y}}}^{-1}$, where Λ_bulk_ is the MFP in bulk and Λ_bdy_ is the MFP shortened by boundary scattering. Since many phonon modes exist in the bulk, the value of Λ_bulk_ depends on the wave vector **q** and phonon branch *s*. Using Λ_eff_ and the bulk properties of phonons, namely the specific heat *C* and group velocity *v*, we obtain the thermal conductivity *κ* in nanostructures by:

1 $$\kappa \,=\,\sum _{{\bf{q}},s}{C}_{{\bf{q}}s}{v}_{{\bf{q}}s}{{\rm{\Lambda }}}_{{\rm{e}}{\rm{f}}{\rm{f}},{\bf{q}}{s}}$$

Thus, the values of *C*, *v*, Λ_bulk_ and Λ_bdy_ are necessary to evaluate the thermal conductivity. The bulk properties of phonons are calculated by anharmonic lattice dynamics with interatomic force constants of silicon. Here anharmonic lattice dynamics is performed with the ALAMODE package^42^ and interatomic force constants are obtained by the Quantum Espresso package^43^. The thermal conductivity of a bulk silicon crystal calculated is 150 Wm^−1^K^−1^ at room temperature, thus the method reproduces well the experimental value^44^.

In order to obtain Λ_bdy_, we perform ray tracing simulations^38^. The method consists in statistically calculating the phonon transmission probability *f*_12_ through the simulated system. Phonons are injected on one side of the system with a polar angle *θ*, following which we evaluate their probability of reaching the opposite side of the system at a distance *L*. Phonon reflections at the surfaces of the nanostructure are assumed to be diffuse. Then, Λ_bdy_ can be obtained as^28,38^:

2 $${{\rm{\Lambda }}}_{{\rm{b}}{\rm{d}}{\rm{y}}}=\frac{3}{2M}\cdot L{\int }_{0}^{\pi /2}{f}_{12}(\theta )\cos \theta \sin \theta d\theta $$

where *M* is a correction factor^28^ accounting the effective cross-section area of fishbone NWs calculated by Comsol Multiphysics. Since this method underestimates Λ_bdy_ when *L* is short, we use 100 unit cells of the fishbone NW for our calculations.
